# Testcross performance and combining ability of early maturing maize inbreds under multiple-stress environments

**DOI:** 10.1038/s41598-019-50345-3

**Published:** 2019-09-24

**Authors:** Benjamin Annor, Baffour Badu-Apraku, Daniel Nyadanu, Richard Akromah, Morakinyo A. B. Fakorede

**Affiliations:** 1 CSIR- Crops Research Institute, P. O. Box 3785, Kumasi, Ghana; 2 Department of Crop and Soil Sciences, Faculty of Agriculture, Kwame Nkrumah University of Science and Technology, Kumasi, Ghana; 3 International Institute of Tropical Agriculture (UK) Limited, 7th floor of Grosvenor House, 125 High Street, Croydon, CR0 9XP UK; 4 Cocoa Research Institute of Ghana, P. O. Box 8, Akim Tafo, Ghana; 5 Department of Crop Production and Protection, Obafemi Awolowo University, Ile-Ile, Nigeria

**Keywords:** Plant breeding, Abiotic, Biotic

## Abstract

Availability of multiple-stress tolerant maize is critical for improvement in maize production in West and Central Africa (WCA). A study was carried out to (i) assess a set of inbred lines for combining ability under stressed and optimal conditions, (ii) determine the performance of the testcrosses under different conditions, and (iii) identify outstanding hybrids across the conditions. Two hundred and five testcrosses were planted with five hybrid checks under *Striga-*infested, low soil nitrogen, drought and optimal conditions between 2015 and 2016 in Nigeria. The grain yield inheritance under optimal condition was largely regulated by additive gene effect whereas non-additive gene effects largely regulated grain yield under the three stresses. Four of the inbreds had significant positive general combining ability effects each under low N and drought, and three under *Striga* infestation for grain yield. The inbreds could be vital sources of beneficial alleles for development and improvement of tropical yellow maize hybrids and populations. Hybrids TZEI 443 x ENT 13 and TZEI 462 x TZEI 10 were high yielding and stable; they out-performed the three early maturing released hybrids in WCA. The new hybrids should be extensively assessed and released in the sub-region to improve food security.

## Introduction

About 30 million hectares of arable land in sub-Saharan Africa (SSA) is devoted to maize (*Zea mays* L.) production^1^. Nevertheless, the 1.8 t ha^−1^ average annual yield in the sub-region is not comparable to that of China and the United States with an annual grain yields of 5.6 and 9.7 t ha^−1^, respectively^2^. The very low grain yield of maize in WCA is partly caused by the increased levels of biotic and abiotic stresses^3,4^ such as drought (40 to 90% yield loss at flowering and grain filling)^5,6^, low N (50% annual yield loss)^7^ and *Striga* infestation (30 to 100% yield loss)^8–10^. The three stresses have often resulted in total crop failure on farmers’ fields especially when the three simultaneously occur in the same farm. Thus, there is a strong demand for maize varieties that are nitrogen-use efficient, drought tolerant with improved resistance to *Striga* infestation for enhanced maize productivity and production in SSA. Currently, farmers in *Striga* prone agro-ecological zones of SSA are not willing to accept maize hybrids that are without combined resistance to multiple stresses^11,12^. Development and deployment of maize hybrids with early maturity (90–95 days) and tolerance to low N, *Striga* and drought is therefore very important for sustainable maize production in WCA. Single-cross hybrids are produced from maize inbreds which are usually extracted from source populations, varieties or bi-parental crosses involving two inbred lines and possessing beneficial stress tolerance alleles after several generations of repeated self-pollination. Through this procedure, the stress tolerance alleles from source populations and bi-parental crosses are fixed in the newly developed inbred lines from the International Institute of Tropical Agriculture (IITA). The new inbred lines derived this way are then crossed in hybrid combinations to produce stress tolerant/resistant hybrids which are usually higher yielding than the open pollinated varieties, source populations or bi-parental crosses from which they were developed. Thus, hybrids are normally higher yielding and more uniform than the open-pollinated varieties.

Determining the combining ability (CA) of new inbred lines is beneficial to hybrid maize breeding programs particularly in most of the SSA countries where such programs are still in the infantile stage. In addition to identifying the good combining inbreds in hybrid production, the CA helps breeders to choose breeding strategy most effective for developing hybrids targeted to stress and optimal conditions. Studies on CA of inbred lines under different conditions have been conducted by many workers in SSA^11–18^. However, it is extremely important for successful hybrid programs to assess the CA of all new inbred lines developed or imported. Similarly, research on the CA of maize inbreds and the gene effects modulating most agronomic traits of maize under *Striga* infestation, low N, optimal and drought conditions is also at the infantile stage and results obtained so far have often been contradictory. For example, several independent studies^11,19–21^ indicated that non-additive gene effects influenced grain yield inheritance under drought in contrast to the results of the studies by Amegbor *et al*.^18^, Betrán *et al*.^22^, Meseka *et al*.^23^, Derera *et al*.^24^ Musila *et al*.^25^ and Badu-Apraku *et al*.^26^ in which additive genetic effects influenced grain yield inheritance in tropical maize inbred lines assessed under drought condition. Similar distinct report has been made for maize in CA studies under low N. For instance, other researchers^12,18,27–32^ found under low N dominance of additive gene effects in the grain yield inheritance whereas several other workers^22–24,33–35^ showed that non-additive gene effect was the most important. Under *Striga* infestation, Yallou *et al*.^36^ and Ifie *et al*.^37^, revealed that the inheritance of number of emerged *Striga* plants and *Striga* damage under *Striga* infestation was largely regulated by non-additive gene effects. In contrast, additive genetic effect was found to be more important for *Striga* damage while non-additive gene effect regulated the number of emerged *Striga* plants^38–40^.

The contradicting reports on the CA or gene effect controlling maize yield under *Striga* infestation, low N and drought in the diverse studies could be ascribed to the variations in the inbred lines used. Hence, the need to assess the CA of new inbred lines that are extracted from different germplasm sources so that they could be effectively used for planning crosses to develop synthetic varieties and hybrids with good performance under distinct environmental conditions in WCA. The general aim of the study was to appraise newly developed yellow endosperm maize inbreds which have the ability to mature early under low soil N, drought, *Striga*-infested, optimal and across the four environmental conditions. Specifically, the study was set up to (i) study the performance of early maturing hybrids across multiple-stress as well as optimal (absence of *Striga*, low soil N and drought) conditions and (ii) identify stable and high yielding hybrids across stress and optimal conditions.

## Results

### Analysis of variance in stressed and optimal conditions

Significant variances (mean squares) (*P* < 0.05) of hybrids (G), environments (E), and hybrids x environments interactions (GEI) revealed for almost all the traits measured under low N and drought (Table 1), optimal and *Striga*-infested (Table 2) and across the environmental conditions (Table 3). Hybrid x environment interaction mean squares were significant for all traits with the exception of stay-green characteristic in drought condition, number of ears per plant and plant aspect under low N, and under *Striga* infestation for number of emerged *Striga* plants at 10 WAP. Almost all the measured traits under low N and drought also varied for GCA-tester, GCA-line and SCA (Table 1), *Striga*-infested and optimal (Table 2) and across the environmental conditions (Table 3). Significant mean squares of GCA-tester x E, GCA-line x E interactions were also detected for almost all the traits in low N, drought (Table 1), *Striga*-infested and optimal (Table 2) and across the environmental conditions (Table 3). In the optimal and across the environmental conditions, significant variances of the interactions of SCA x E were obtained for grain yield and almost all the other traits. In contrast, the variances of the interactions of SCA x E in the low N, drought (Table 1), and *Striga*-infested conditions (Table 2) were not significant for almost all the traits.

**Table 1:** Mean squares of different traits of early maturing yellow maize hybrids assessed under three drought and three low N environments in 2015 2016.

Source	DF	YIELD	POLLEN	DYSK	ASI	PLHT	PASP	EASP	EPP	STGR
**Drought**										
Env	2	933552083**	2086.49**	6098.90**	1021.91**	53273.71**	258.97**	106.02**	6.51**	338.22**
Rep (Env)	3	20086749**	195.90**	296.77**	24.70**	5387.75**	11.84**	9.69**	0.10*	45.91**
Block (Env*Rep)	78	2154489**	9.75**	11.11**	2.38	1016.06**	1.74**	1.54**	0.05**	2.23**
Hybrid	209	2353347**	15.41**	19.15**	3.72**	1294.89**	1.35**	1.48**	0.05**	1.78**
GCA-line	40	3598950**	39.18**	52.83**	5.48**	1052.83**	1.64**	2.17**	0.06**	3.29**
GCA-tester	4	12314941	174.31**	159.24**	25.88**	41254.00**	10.64**	10.60**	0.08	12.51**
SCA	160	1808932**	7.92**	9.51	2.98	454.15	0.95	1.11*	0.05*	1.33
Env*Hybrid	418	1248052**	7.07**	9.55**	3.47**	293.68	0.98**	0.91**	0.04**	1.43
Env*GCA-line	80	1500763**	13.63**	15.74**	4.30**	335.88	1.27*	1.53**	0.05*	1.66
Env*GCA-tester	8	8997020**	14.19**	37.24**	15.62**	1863.65**	3.36**	4.89**	0.20**	4.24**
Env*SCA	320	1174788	6.72*	8.71	3.12*	363.44	0.93	0.80	0.04	1.55
Error	614	1034603	5.48	8.40	2.58	387.33	0.90	0.86	0.04	1.51
**Low soil nitrogen**										
Env	2	469014141**	442.25**	90.19**	195.57**	501625.38**	35.92**	5.01**	2.36**	885.74**
Rep (Env)	3	8815749**	10.96**	25.60**	9.23**	3413.71**	7.89**	2.31**	0.33**	5.64**
Block (Env*Rep)	78	768071**	3.53**	3.45**	0.57	560.14**	1.20**	0.98**	0.02**	1.53**
Hybrid	209	1720841**	14.57**	15.41**	1.21**	924.39**	1.26**	0.98**	0.02**	0.96**
GCA-line	40	3055246**	34.11**	41.33**	2.87**	1002.56**	1.36**	1.14**	0.04**	1.27**
GCA-tester	4	10821013**	200.27**	184.11**	2.76**	28223.65**	23.39**	10.37**	0.15**	8.59**
SCA	160	1479947**	7.11**	6.71**	0.92**	391.09**	1.02**	0.91**	0.02	0.89**
Env*Hybrid	418	753036**	3.54**	3.65**	0.62*	416.24**	0.46	0.48**	0.02	0.69**
Env*GCA-line	80	1135984**	6.84**	8.04**	1.11**	505.95**	0.66	0.61*	0.02	1.09**
Env*GCA- tester	8	4985611**	8.80**	7.54**	1.69**	6639.60**	1.25*	3.83**	0.06**	3.30**
Env*SCA	320	680550**	3.20**	3.04**	0.58	282.16	0.48	0.43	0.02	0.74**
Error	614	396887	1.75	1.94	0.56	282.36	0.53	0.47	0.02	0.59

*^,^**Significant at 0.05 and 0.01 probability levels, respectively; Env = environment; Rep = replication; YIELD = Grain yield (kg ha^−1^); Pollen = days to 50% anthesis; DYSK = days to 50% silking; ASI = anthesis-silking interval; PLHT = plant height (cm); PASP = plant aspect; EASP = ear aspect; EPP = ears per plant; STGR = stay-green characteristic.

**Table 2:** Mean squares of different traits of early maturing yellow maize hybrids planted under four *Striga*-infested and seven optimal environments in 2015 and 2016.

Source	DF	YIELD	POLLEN	DYSK	ASI	PLHT	EASP	EPP	SDR8	SDR10	ESP8	ESP10
***Striga*** **-infested environments**												
Env	3	320119378**	942.34**	1142.62**	34.24**	190066.61**	45.53**	1.05**	113.07**	18.99**	331.12**	347.02**
Rep (Env)	4	6309005**	57.53**	63.32**	2.59	426.16	3.72**	0.07**	2.50**	2.55**	5.49**	1.30**
Block (Env*Rep)	104	3503982**	7.45**	15.66**	3.58**	710.50**	1.20**	0.03**	1.77**	1.50**	1.12**	4.52**
Hybrid	209	2678317**	17.63**	25.84**	3.36**	732.76**	1.05**	0.05**	1.05**	1.22**	1.15**	0.95**
GCA-line	40	5193282**	39.82**	60.80**	7.14**	810.67**	1.83**	0.10**	2.48**	3.01**	1.02*	1.37**
GCA-tester	4	15636636**	286.88**	425.17**	24.91**	8360.67**	4.12**	0.68**	9.99**	8.15**	13.15**	11.74**
SCA	160	2151012**	8.43**	12.12**	2.77*	587.13**	0.84**	0.03**	0.70	0.88**	1.10**	0.83**
Env*Hybrid	627	1437116**	5.25**	7.98**	2.34**	518.62**	0.66**	0.02**	0.63**	0.74**	0.66*	0.55
Env*GCA-line	120	2187966**	8.49**	11.98**	2.35	502.56**	0.92**	0.03**	0.88**	1.23**	0.90**	0.75
Env*GCA- tester	12	11183847**	11.19**	29.38**	7.27**	2042.02**	3.32**	0.14**	2.93**	5.28**	1.82**	0.68
Env*SCA	480	1295966	5.67**	8.31**	2.43	509.22**	0.64	0.02	0.66	0.68	0.73	0.61
Error	819	1311421	3.48	6.57	2.15	360.38	0.58	0.02	0.64	0.67	0.66	0.61
**Optimal environments**												
**Source**									**PASP**			
Env	6	387535840**	1011.15**	1086.44**	41.71**	166127.89**	14.74**	1.35**	49.03**			
Rep(Env)	7	15534957**	30.99**	37.65**	0.79	2376.10**	3.60**	0.09**	0.57			
Block(Env*Rep)	182	2197072**	4.01**	4.80**	0.52	470.32**	0.83**	0.03**	0.82**			
Hybrid	209	10921000**	31.09**	45.36**	2.47**	1463.83**	3.21**	0.04**	3.76**			
GCA-line	40	18278629**	122.93**	196.62**	9.33**	1722.24**	4.09**	0.05**	5.89**			
GCA-tester	4	224468759**	261.14**	275.63**	4.69**	49584.85**	73.22**	0.07**	91.72**			
SCA	160	4393834**	6.35**	7.11**	0.95**	470.95**	1.49**	0.04**	1.27**			
Env*Hybrid	1254	1561670**	4.31**	5.77**	0.66**	350.76**	0.53**	0.03**	0.50**			
Env*GCA-line	240	2534630**	13.35**	19.88**	1.25**	446.93**	0.75**	0.03**	0.68**			
Env*GCA- tester	24	3485354**	13.75**	11.29**	0.90**	3966.25**	2.10**	0.06**	1.52**			
Env*SCA	960	1481235**	2.67**	3.10**	0.55*	292.22**	0.52*	0.03**	0.51**			
Error	1434	1031410	1.82	2.27	0.49	203.04	0.46	0.02	0.41			

*^,^**Significant at 0.05 and 0.01 probability levels, respectively; Env = environment; Rep = replication; YIELD = Grain yield (kg ha^−1^); Pollen = days to 50% anthesis; DYSK = days to 50% silking; ASI = anthesis-silking interval; PLHT = plant height (cm); PASP = plant aspect EASP = ear aspect; EPP = ears per plant; SDR8 and SDR10 = *Striga* damage rating at 8 and 10 WAP; ESP8 and ESP10 = number of emerged *Striga* plants at 8 and 10 WAP.

**Table 3:** Mean squares of different traits of early maturing yellow maize hybrids across four management conditions in 2015 and 2016.

Source	DF	YIELD	POLLEN	DYSK	ASI	PLHT	EASP	EPP	DF	PASP	DF	STGR
Env	16	848804140**	1862.51**	3625.96**	411.37**	182027.03**	37.50**	2.53**	12	92.43**	5	506.67**
Rep (Env)	17	12981660**	62.80**	87.29**	6.92**	2631.86**	4.48**	0.13**	13	4.86**	6	25.77**
Block (Env*Rep)	442	2244889**	5.75**	8.23**	1.58**	638.99**	1.07**	0.03**	338	1.12**	156	1.88**
Manag-c	3	2496720203**	5282.93**	11896.90**	1264.64**	78555.72**	50.98**	3.82**	2	112.64**	1	85.43**
Hybrid	209	12033989**	62.47**	83.04**	4.74**	3119.98**	4.17**	0.05**	209	4.87**	209	1.77**
GCA-line	40	19326650**	208.43**	306.88**	15.97**	3076.65**	4.66**	0.09**	40	6.71**	40	3.05**
GCA-tester	4	188137337**	880.46**	956.38**	24.76**	116078.79**	79.47**	0.39**	4	117.24**	4	14.61**
SCA	160	6078917**	13.34**	15.58**	1.95**	771.63**	2.30**	0.04**	160	1.83**	160	1.36*
Manag-c*Hybrid	627	2048507	5.93	8.08	2.05*	461.02	0.91**	0.04**	418	0.81	209	1.01
Env*Hybrid	3344	1459456**	4.95**	6.75**	1.57**	398.37**	0.65**	0.03**	2508	0.62**	1045	1.04**
Env*GCA-line	640	2365289**	10.88**	15.47**	2.14**	461.54**	1.01**	0.04**	480	0.84**	200	1.40**
Env*GCA- tester	64	9845809**	12.76**	20.83**	5.95**	3642.15**	3.68**	0.12**	48	2.24**	20	4.31**
Env*SCA	2560	1265050**	4.33**	5.43**	1.48**	356.49**	0.60*	0.03**	1920	0.61*	800	1.09
Error	3484	986270	2.86	4.33	1.27	286.74	0.56	0.02	2664	0.55	1229	1.10

*^,^**Significant at 0.05 and 0.01 probability levels, respectively; Env = environment; Rep = replication; Manag-c = management condition; YIELD = Grain yield (kg ha^−1^); Pollen = days to 50% anthesis; DYSK = days to 50% silking; ASI = anthesis-silking interval; PLHT = plant height (cm); PASP = plant aspect; EASP = ear aspect; EPP = ears per plant; STGR = stay-green characteristic.

### Relative contributions of specific combining ability and general combining ability effects of inbred lines

Under *Striga* infestation, low N and drought conditions, the percentage of the SCA effects was greater than that of the GCA for almost all the traits (Table 4). In contrast, the GCA effects contributed a greater portion of the total genetic effects for most of the other traits measured under optimal and across environmental conditions (Table 4). The GCA effects of almost all the traits measured under low N, drought, optimal, *Striga*-infested and across environmental conditions showed that out of the 41 inbred lines, only the grain yield GCA effects for TZEI 160, TZEI 515, TZEI 443, TZEI 161 were positive and significant. In addition, significantly negative GCA effects were recorded by inbreds TZEI 443, TZEI 432, TZEI 182 and TZEI 474 for stay-green characteristic in the contrasting conditions. However, all the five testers had no significant and positive GCA effects under the four management conditions for grain yield (Table 5). Only TZEI 465, TZEI 462, TZEI 175 and TZEI 486 showed positive and significant GCA effects for grain yield under low N; TZEI 472, TZEI 175 and TZEI 462 under *Striga*-infested environments; TZEI 462, TZEI 124, TZEI 522, TZEI 175, TZEI 443 and ENT 8 under optimal growing conditions; TZEI 462, TZEI 443, TZEI 465, TZEI 522, TZEI 486, TZEI 494, ENT 8, TZEI 16, TZEI 124 and TZEI 175 across the environmental conditions (Table 5). In contrast, the five inbred testers had no positive and significant GCA effects in the contrasting environmental conditions for grain yield except ENT 13 and TZEI 129 under the optimal condition.

**Table 4:** Proportion (%) of total genotypic sum of squares of grain yield and other agronomic traits of early maturing inbred lines attributable to GCA-line, GCA-tester and SCA under drought, low soil N, optimal, *Striga*-infested and across the four management conditions.

Traits	GCA-line	GCA-tester	SCA
**Grain yield**			
Drought	30	10	60
Low soil nitrogen	30	11	59
*Striga* infestation	34	10	56
Optimum	31	39	30
Across	31	30	39
**Anthesis-silking interval**			
Drought	27	13	60
Low soil nitrogen	42	4	54
*Striga* infestation	34	12	54
Optimum	69	3	28
Across	14	2	84
**Plant aspect**			
Drought	25	16	58
Low soil nitrogen	17	30	53
Optimum	29	46	25
Across	28	49	23
**Ear aspect**			
Drought	28	14	58
Low soil nitrogen	20	18	63
*Striga* infestation	33	7	60
Optimum	24	42	34
Across	28	48	23
**Ears per plant**			
Drought	24	3	73
Low soil nitrogen	30	12	58
*Striga* infestation	34	24	42
Optimum	26	3	70
Across	22	9	69
**Stay-green characteristic**			
Drought	33	13	54
Low soil nitrogen	22	15	63
Across	46	22	32
***Striga*** **damage at 8 WAP**			
*Striga* infestation	39	16	45
***Striga*** **damage at 10 WAP**			
*Striga* infestation	41	11	48
**Number of emerged** ***Striga*** **plants at 8 WAP**			
*Striga* infestation	15	19	65
**Number of emerged** ***Striga*** **plants at 10 WAP**			
*Striga* infestation	23	20	56

**Table 5:** General combining ability effects of lines and testers for grain yield under drought, *Striga*-infested, low N and optimal conditions.

Inbred	Grain yield (kg ha^−1^)			
	Drought	Low N	*Striga*-infested	optimal
**Lines**				
TZEI 415	−627.7**	−802.6**	−473.3*	−1244.5**
TZEI 428	−194.3	−475.9*	−84.6	−457.5*
TZEI 430	−239.3	−580.4**	−517.2*	−864.6**
TZEI 432	−684.0**	−493.1*	−394.0	−749.9**
TZEI 433	303.7	−404.5*	−167.5	−132.8
TZEI 439	−386.9	−124.9	−58.1	−324.4
TZEI 441	−397.5	−260.8	152.4	−476.5*
TZEI 442	−321.9	2.9	110.4	−85.0
TZEI 443	751.0**	279.9	395.2	860.5**
TZEI 461	35.0	138.0	8.7	201.7
TZEI 462	235.8	587.6**	759.5**	907.2**
TZEI 464	−143.7	348.3	−176.8	114.4
TZEI 465	−90.6	500.9*	294.3	298.0
TZEI 470	−47.4	−177.7	−64.2	−112.7
TZEI 472	−246.7	−420.9*	474.0*	−205.3
TZEI 474	424.9	−2.3	−106.9	−342.5
TZEI 483	−35.7	−63.5	−164.7	−427.3*
TZEI 484	−86.0	172.6	−205.9	−128.9
TZEI 486	218.4	434.5*	426.0	175.3
TZEI 495	361.5	88.3	−43.3	161.8
TZEI 507	57.4	−263.5	216.4	−346.3
TZEI 508	−280.8	−0.4	341.5	43.6
TZEI 515	519.1*	−78.0	−29.6	−158.9
TZEI 516	94.7	−19.6	−75.5	−144.0
TZEI 518	196.1	173.2	32.8	112.1
TZEI 520	−204.9	28.6	−157.5	183.7
TZEI 522	−123.2	365.7	330.6	735.4**
TZEI 449	−516.2*	130.4	−979.7**	165.5
TZEI 455	−47.0	−366.3	103.1	−709.5**
TZEI 450	−554.1**	−225.2	−501.9*	−381.3*
TZEI 494	355.3	247.0	301.7	327.7
TZEI 124	245.7	221.0	−144.0	833.4**
TZEI 182	135.2	−177.2	−499.6*	−290.7
TZEI 16	24.5	5.6	166.1	760.0**
TZEI 24	155.3	−48.9	372.0	−363.1
TZEI 160	613.7**	−155.3	−340.3	−731.9**
TZEI 161	460.5*	−40.2	14.7	−248.8
TZEI 173	−532.4*	1.5	330.0	342.2
TZEI 175	6.0	513.7**	528.8*	738.2**
ENT 8	313.7	352.5	136.2	794.6**
ENT 17	−174.1	351.0	−745.0**	12.9
SED line	220.9	192.2	231.0	188.0
**Testers**				
TZEI 10	−208.3	141.9	228.4	109.4
TZEI 17	−7.3	−55.2	−121.4	−108.0
TZEI 23	−257.9	−346.7*	−300.0	−1011.3**
TZEI 129	178.9	60.6	−41.0	165.2*
ENT 13	242.5	170.4	180.9	703.3**
SED tester	171.1	127.3	165.2	69.7

### Early maturing yellow maize hybrids performance under different management conditions

The 25 (best 15 and the worst 10) yellow maize hybrids selected using the IITA selection index under low N, drought, optimal, *Striga* and across environmental conditions are shown in Supplementary Tables 3, 4, 5, 6 and 7, respectively. The 25 testcrosses under drought condition had grain yields from 865 kg/ha for TZEI 432 x TZEI 10 to 4967 kg/ha for TZEI 443 x ENT 13, with an average of 2639 kg/ha. Hybrid TZEI 443 x ENT 13 had the highest yield under drought and produced 4967 kg/ha as compared with the 3500 kg/ha achieved by the best released check-TZEI 124 x TZEI 25, 42% yield advantage (Supplementary Table 3). The reduction in grain yield in the drought condition compared to the optimal ranged from 17 to 75% with an average of 45% in the drought condition. Days to 50% anthesis for the 15 drought tolerant hybrids ranged from 52 to 57 while days to 50% silking was from 54 to 60. Generally, increase in days to silking and the days to anthesis lead to an increase in the anthesis-silking interval, poor plant aspect, fewer ears per plant and ear aspect, accelerated senescence of leaves and high yield reduction among the testcrosses that produced poor grain yield and had negative selection indices (selection index) under drought condition (Supplementary Table 3).

Grain yield under low N was from 1469 kg/ha for TZEI 182 x TZEI 10 to 4096 kg/ha for TZEI 443 x ENT 13 with an average of 2704 kg/ha. The highest performing hybrid in terms of yield (TZEI 443 x ENT 13) under low N was not statistically different from the top seven high yielding and low N tolerant hybrids. However, it obtained higher yield and low N tolerance compared to the five checks. The released check which had high yield and low N tolerance was out-yielded by the best hybrid by 20%. Between 31 to 61% yield reduction was obtained under low N condition compared to that under optimal with an average of 44%. Days to 50% anthesis for the 15 low N tolerant hybrids ranged from 50 to 54 while days to 50% silking was from 51 to 54 (Supplementary Table 4).

Grain yield under *Striga* infestation ranged from 1333 kg/ha for TZEI 455 x TZEI 23 to 4799 kg/ha for the released hybrid (TZEI 11 x TZEI 24) with an average of 3124 kg/ha (Supplementary Table 8). The check which had high yield under *Striga* infestation (TZEI 11 x TZEI 24) was not different from the 5 top-yielding hybrids out of the 15 *Striga* resistant hybrids as well as the released check-TZEI 124 x TZEI 25. The reduction in yield under the *Striga*–infested condition compared to that of optimal was from 2 to 57% with an average of 35%. Days to 50% anthesis for the 15 best *Striga* resistant hybrids ranged from 52 to 56 while days to 50% silking was from 53 to 59 (Supplementary Table 5).

In the optimal conditions, the yield varied from 2428 kg/ha for TZEI 455 x TZEI 23 to 7260 kg/ha for the released (commercial) hybrid check, TZEI 124 x TZEI 25 with an average of 4904 kg ha^−1^. Conversely, the yield of the check in the optimal condition was not different from the best multiple-stress tolerant hybrid, TZEI 443 x ENT 13 (Supplementary Table 6).

Presented in Supplementary Table 7 are the 15 hybrids with multiple-stress tolerance and the 10 susceptible and five checks across management conditions. The yield varied from 1897 kg/ha for TZEI 455 x TZEI 23 to 5535 kg/ha for TZEI 443 x ENT 13 with an average of 3697 kg/ha. Hybrid, TZEI 443 x ENT 13 had yield which was significantly higher than those of the other hybrids across management condition.

**Figure 1:**
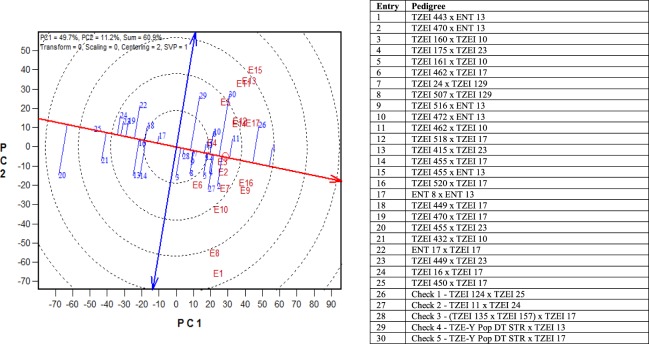
An entry/tester genotype main effect plus genotype x environment biplot of grain yield of 25 selected early maturing hybrids evaluated across drought, low N, *Striga*-infested and optimal environments in 2015 and 2016. E1 = Minjibir under drought and heat stress, 2014/2015; E2 = Ikenne under drought, 2015/2016; E3 = Kadawa under drought, 2016; E4 = Mokwa under low N, 2015; E5 = Ile-Ife under low N, 2015; E6 = Mokwa under low N, 2016; E7 = Mokwa under *Striga*-infested, 2015; E8 = Abuja under *Striga*-infested, 2015; E9 = Abuja under *Striga*-infested, 2016; E10 = Mokwa under *Striga*-infested, 2016; E11 = Mokwa under optimal, 2015; E12 = Mokwa under optimal, 2016; E13 = Ikenne under optimal, 2015; E14 = Ikenne under optimal, 2016; E15 = Abuja under optimal, 2015; E16 = Abuja under optimal, 2016; E17 = Ile-Ife under optimal, 2015.

The GGE biplot “stability vs. mean performance” for grain yield of the selected 25 testcrosses and five checks across the four management conditions is indicated in Fig. 1. The entry/tester GGE biplot displayed TZEI 462 x TZEI 10 (11) and TZEI 443 x ENT 13 (1) as the highest yielding with relatively high stability. In contrast, hybrids TZEI 516 x ENT 13 (9), TZEI 24 x TZEI 129 (7), TZEI 455 x ENT 13 (15), TZEI 518 x TZEI 17 (12), and the check-(TZEI 135 x TZEI 157) x TZEI 17 (28) had the greatest stability across the management conditions. Even though the yields were higher than the average yield, the yields were relatively low. The released hybrid, TZEI 124 x TZEI 25 (26) had the second highest grain yield but was highly unstable. On the contrary, hybrids 21 (TZEI 432 x TZEI 10), 20 (TZEI 455 x TZEI 23) and 25 (TZEI 450 x TZEI 17) had the lowest yield across the management conditions.

## Discussion

One important concern of plant breeders and agronomists when conducting studies similar to those reported here is to ensure the effectiveness of the stress under investigation. One way to do that is to compare the performance under stress with that under non-stress conditions within otherwise similar environmental conditions. The study showed that the stresses were quite effective. Relative to the non-stress conditions, drought reduced grain yield by 45%, low N by 44% and *Striga* infestation by 35% on the average; all of which were equal with or even higher than the reductions reported in earlier studies on drought stress^5,24,26,41^, low N^42^, and *Striga* infestation^26^. The methodology used for imposing each stress, conclusions reached, and the stress tolerant hybrids selected in this study are, therefore, reliable and may be used as basis for further studies on the improvement of maize productivity for SSA.

The best of the 205 new hybrids evaluated in the study consistently out-yielded the best available hybrids used as checks for each and across environments. Hybrid TZEI 443 x ENT 13, the most drought tolerant hybrid, yielded more than the best released hybrid, Sammaz 41 or Tamalaka (TZEI 124 x TZEI 25) by 42% under drought. Hybrid TZEI 443 x ENT 13 was also the highest low N tolerant and highest yielding under low N; it out-yielded the low N tolerant and high yielding released hybrid, Kunjor-wari (TZE-Y Pop DT STR x TZEI 17) by 20%, an indication that significant gain has been achieved in breeding for early maturing hybrids with high grain yield and low N tolerance in SSA. Hybrid TZEI 443 x ENT 13 therefore has the potential of producing high yield in low N soils, an important maize production constraint which is against increased maize production in SSA. In the *Striga*-infested condition, yields of the top five *Striga* resistant hybrids, TZEI 24 x TZEI 129, TZEI 472 x ENT 13, TZEI 462 x TZEI 17, TZEI 443 x ENT 13 and TZEI 462 x ENT 13 were equal to that of the check, TZEI 11 x TZEI 24. The hybrids therefore have the potential to contribute to increased grain yield in *Striga* prone areas of WCA. The major aim of maize research in WCA is to develop hybrids with tolerance or resistance to low N, *Striga* infestation and drought combined since most of the time occur on farmers’ fields together. It is striking that the early maize hybrids, TZEI 470 x ENT 13, TZEI 443 x ENT 13 and several others, showed outstanding performance under multiple stresses. These hybrids have the potential to contribute to increased maize productivity and improve food security in the sub-region.

Statistically significant GCA (estimate of additive gene action), SCA (dominance gene action) and their interactions with the environment (additive x environment and dominance x environment gene action) occurred in this study, but varied among the environmental conditions. Under drought, days to anthesis, plant height, days to silking and ear height were inherited by additive rather than dominant gene action. However, the dominating effect of the grain yield, ear and plant aspects, EPP ASI, and the stay-green trait SCA sum of squares over that of GCA under drought showed that the inheritance of the traits was regulated by the non-additive gene effects and that SCA was the main constituent which caused the variations observed within the hybrids. These results contradicted the earlier reports of Badu-Apraku *et al*.^11^, Betrán *et al*.^22^, Meseka *et al*.^23^, Derera *et al*.^24^ and Oyekunle and Badu-Apraku^43^ that additive gene effects mainly controlled the inheritance of yield under drought conditions. Conversely, the result validated that of Souza *et al*.^20^ and Badu-Apraku *et al*.^26^ who revealed that non-additive gene effects regulated yield under drought conditions.

Under low N, the inheritance of days to silking, plant height, days to anthesis, root lodging, ear height and ear rot was largely attributable to additive gene effects while that of grain yield, ASI, stalk lodging, plant aspect, husk cover, ear aspect, EPP and the stay-green characteristic were largely modulated by non-additive gene effects. These results are inconsistent with that of several other workers^11,16,29,30,32,36,44^, who revealed that additive genetic action largely influenced the inheritance of almost all the measured traits under low N environments. However, the result confirmed that of Badu-Apraku *et al*.^15^, Betràn *et al*.^22^, Meseka *et al*.^23^, Derera *et al*.^24^, Makumbi *et al*.^34^, Ndhlela^45^, Meseka *et al*.^46^, and Mafouasson^47^ who found preponderance of non-additive gene effects over additive genetic action for yield in low N conditions.

Under *Striga* infestation, additive genetic action was found to be the most influential in the inheritance of days to 50% anthesis, EPP, and *Striga* damage whereas non-additive genetic action was the major contributor to the variations observed for grain yield, ear aspect, plant height, ASI, and the number of emerged *Striga* plants. This does not agree with the results of Badu-Apraku *et al*.^16^, Ifie *et al*.^37^ and Akinwale *et al*.^48^ who found a dominating effect of the sum of squares of GCA over that of SCA for yield under *Striga* infestation. Moreover, the result is in disagreement with Yallou *et al*.^36^ and Ifie *et al*.^37^, who found that non-additive gene effects mainly influenced the inheritance of both the number of emerged *Striga* plants and *Striga* damage. On the contrary, the present results agreed with those of Kim^38^, Akanvou *et al*.^39^ and Badu-Apraku^40^ who showed that additive genetic action mainly influenced the inheritance of *Striga* damage while non-additive gene effect largely influenced the number of emerged *Striga* plants.

The high GCA recorded over SCA sum of squares for all the traits with the exception of ear rot and EPP under optimal environments showed that additive genetic action principally influenced the inheritance of most of the traits than non-additive. The finding is in accordance with that of Katsantonis *et al*.^33^, Makumbi *et al*.^34^ and Mafouasson^47^ who reported that additive genetic action influenced the inheritance of maize yield under optimal condition and that almost all the traits could be improved using the recurrent selection procedure. The major effect of GCA over SCA sum of squares suggested that, testing of early generation progenies may be successful. It also implied that promising yellow endosperm maize hybrids can be selected principally based on the GCA effects as revealed by Baker^49^. However, Rizzi *et al*.^27^, Below *et al*.^29^ and Kling *et al*.^30^ revealed that non-additive genetic actions were accountable for the differences in maize yield under optimal condition. The differences in the findings of this study and that of some earlier researchers may be ascribed to the variations in the inbred lines used as well as the climatic and soil conditions.

The positive and significant GCA effects recorded for some early yellow inbreds for the yield in low N, *Striga*-infested, optimal and drought conditions showed that those inbred lines would contribute improved yield in their offspring under the contrasting management conditions.

The positive and significant GCA effects of grain yield obtained for inbred lines TZEI 465, TZEI 462, TZEI 175 and TZEI 486 under low N; TZEI 472, TZEI 175 and TZEI 462 under *Striga-*infested environments; TZEI62, TZEI 124, TZEI 522, TZEI 175, TZEI 443 and ENT 8 under optimal growing conditions; TZEI 462, TZEI 443, TZEI 465, TZEI 522, TZEI 486, TZEI 494, ENT 8, TZEI 16, TZEI 124 and TZEI 175 across the environmental conditions suggested that these inbred lines could serve as sources of beneficial alleles for improving tropical germplasms for high grain yield. Furthermore, the significantly negative GCA effects recorded for TZEI 443, TZEI 432, TZEI 182 and TZEI 474 for stay green characteristic under low N and drought environments.

## Methods

### Genetic materials and generation of testcrosses

Based on their responses to *Striga* infestation and drought, 41 early maturing yellow endosperm maize inbred lines were selected and crossed to each of the five elite inbred testers (TZEI 10, TZEI 23, TZEI 17, ENT 13 and TZEI 129) using the line x tester mating design. The five elite inbred testers were regarded as males and crossed to the 41 early maturing yellow endosperm maize inbred lines as females to develop 205 testcross hybrids.

### Field experiments

The 205 testcross hybrids plus five checks [including three commercial hybrids (TZEI 124 x TZEI 25 and TZE-Y Pop DT STR x TZEI 13 released in Mali and Nigeria; TZE-Y Pop DT STR x TZEI 17 released in Ghana)] were assessed under optimal (non-stressed) and three stressed (low N, *Striga* infestation and drought) conditions. Each entry was planted under optimal condition during the 2015 and 2016 rainy seasons at Mokwa, Ikenne, Ile-Ife and at Abuja during the 2015 rainy season (Supplementary Table 2). The agronomic and management practices adopted for the experiments have been reported by Badu-Apraku *et al*.^50^.

Low N (30 kg/ha) experiments were carried out at Mokwa during the rainy seasons of 2015 and 2016 and Ile-Ife during the 2015 rainy season (Supplementary Table 2). Details on how the low soil N environment was achieved at Mokwa and Ile-Ife and the management practices observed have been described by Badu-Apraku^50^.

Also, the hybrids were established under managed drought during the dry season at Minjibir in 2015 and Ikenne in 2016. The other drought trial was carried out at Kadawa during the rainy season of 2016 (Supplementary Table 2). The drought experiments conducted at Minjibir and Ikenne were as described by Badu-Apraku *et al*.^50^. However, due to the high temperature at Minjibir during the experiment period (February–March 2015) the irrigation was resumed at two weeks after flowering to enable completion of grain filling.

Each hybrid was also planted under artificial *Striga* infestation at Mokwa and Abuja in 2015 and 2016 (Supplementary Table 2). The soil on the *Striga*-infested field was injected with ethylene gas at two weeks before planting on the *Striga* fields. This was done to induce any surviving *Striga* seeds to forcefully germinate and die subsequently. The *Striga* infestation was done based on the method of Kim^51^. Detailed description of the management of the *Striga*-infested fields has been provided by Badu-Apraku *et al*.^50^.

All trials were carried out using a 14 × 15 alpha lattice in two replications. Each hybrid was assessed in a single-row plot, 3 m long spaced 0.40 m between hills and 0.75 between rows. Three seeds were initially planted in each planting hole and after at 2 WAP the germinated seeds were thinned to two to obtain a population density of approximately 66,666 plants ha^−1^.

### Data collection

Days to 50% anthesis (DA), plant height, days to 50% silking (DS) and ear aspect were recorded in all experiments. Anthesis-silking interval (ASI) was calculated as the difference between DA and DS while the number of maize ears per plant was estimated as the number of harvested ears per plot divided by the number of available plants. Plant aspect was recorded for the low N, optimal and drought plots on a scale of 1 (excellent) −9 (poor) based on overall plant type. Stay-green characteristic was recorded for the low N and drought experiments at 70 days after planting (DAP) as described by Annor and Badu-Apraku^17^. The number of emerged *Striga* plants and *Striga* damage were scored at 56 and 70 DAP (8 and 10 weeks after planting (WAP)) in the *Striga*-infested fields as proposed by Kim^51^. Under optimal and *Striga-*infested conditions, all the harvested and de-husked ears from each plot were weighed after which samples were shelled to obtain the moisture content (%) of the grains. A shelling percentage of 80% was assumed and yield adjusted to 15% moisture content was calculated from the ear weight and converted to kg/ha. For experiments conducted under low N and drought environments, ears harvested from each plot were shelled, the grains were weighed, and samples taken to measure the percentage grain moisture. Grain yield (kg/ha) which was adjusted to 15% moisture content was estimated from the grain weight after shelling.

### Statistical analysis

Analysis of variance (ANOVA) were performed individually for data collected under each management conditions (low N, drought, optimal and *Striga*) with mixed model procedure in SAS^52^. Combined ANOVA was performed thereafter across the 17 test environments (locations-management combinations). In all analyses, environments, replicates within environment and blocks within replicates × environment interaction were observed as random factors while the hybrid was made a fixed factor. The least square means were estimated using SAS and separated using standard error (S.E).

The means generated from the ANOVA under the four management conditions and combined across all 17 environments were used for the line x tester analysis as reported by Comstock and Moll^53^ and Singh and Chaudhary^54^. The specific combining ability (SCA) and general combining ability (GCA) effects as well as their standard errors were estimated for all traits under the different management conditions using SAS software version 9.3.

The relative importance of GCA (GCA-tester + GCA-line) and SCA effects was estimated as the proportion (percentage) of the genetic sum of squares of a trait due to SCA or GCA^55,56^. The greater the proportion of the sum of square of a trait that is due to GCA or SCA, the greater was the predictability of the trait based on GCA or SCA^49^.

A selection index (I) for identifying and selecting the best performing hybrids under low N and drought conditions was estimated as proposed by Badu-Apraku *et al*.^4^ whereas the base index used for hybrids evaluated under *Striga* infestation was calculated as described by MIP^57^ and Menkir and Kling^58^. To identify hybrids with combined tolerance or susceptibility to all three stressed environments (low N, *Striga* and drought), a multiple-trait selection index (MI) was estimated as explained by Badu-Apraku *et al*.^21^ and Amegbor *et al*.^18^.

Based on the MI, the top 15 low-N, *Striga* and drought tolerant/resistant and the ten most susceptible as well as the five checks were selected for genotype main effect plus genotype × environment interaction (GGE) biplot analysis to breakdown the G x E interactions into its component parts^59^. The GGE biplot was used to identify the most promising hybrids with high and stable yield across stressed and optimal environments^59^.

## Supplementary information

Supplementary Tables

## Data Availability

The data that support the findings of this study are available from the corresponding author upon request.
