# Supplementary material for: Testcross performance and combining ability of early maturing maize inbreds under multiple-stress environments
Source: Sci Rep. 2019 Sep 24;9:13809. doi: 10.1038/s41598-019-50345-3 (PMC6760492; doi:10.1038/s41598-019-50345-3)
Supplement: Supplementary file 1 — Supplementary Tables [file 41598_2019_50345_MOESM1_ESM.doc]

**Testcross performance and combining ability of early maturing maize inbreds under multiple-stress environments**

Benjamin Annor^1, 2^, Baffour Badu-Apraku^*3^, Daniel Nyadanu^4^, Richard Akromah^2^, and Morakinyo A. B. Fakorede^5^

^1^CSIR- Crops Research Institute, P. O. Box 3785, Kumasi, Ghana

^2^Department of Crop and Soil Sciences, Faculty of Agriculture, Kwame Nkrumah University of Science and Technology, Kumasi, Ghana

^3^International Institute of Tropical Agriculture (UK) Limited, 7th floor of Grosvenor House, 125 High Street, Croydon CR0 9XP UK, Tel.: +1 201 6336094, Fax.: +44 (208) 711 3786 (via UK)

^4^Cocoa Research Institute of Ghana, P. O. Box 8, Akim Tafo, Ghana

^5^Department of Crop Production and Protection, Obafemi Awolowo University, Ile-Ile, Nigeria

*Corresponding Author (Email: b.badu-apraku@cgiar.org)

Supplementary Table 1. Description of early maturing maize inbreds used for the production of the testcrosses.

| S/N | Inbred | Designation | Source | Reaction to drought | Reaction to *Striga* |
| --- | --- | --- | --- | --- | --- |
| 1 | TZEI 415 | (TZEI 11 x TZEI 8) S6 inb 18-1/3-1/2-1/1 | IITA | Tolerant | Tolerant |
| 2 | TZEI 428 | (TZEI 11 x TZEI 8) S6 inb 30-3/3-7/9-1/1 | IITA | Tolerant | Tolerant |
| 3 | TZEI 430 | (TZEI 11 x TZEI 8) S6 inb 37-1/3-3/3-1/1 | IITA | Tolerant | Susceptible |
| 4 | TZEI 432 | (TZEI 11 x TZEI 8) S6 inb 47-3/4-4/11-1/1 | IITA | Tolerant | Susceptible |
| 5 | TZEI 433 | (TZEI 11 x TZEI 8) S6 inb 81-1/4-8/10-1/1 | IITA | Tolerant | Susceptible |
| 6 | TZEI 439 | (TZEI 11 x TZEI 8) S6 inb 92-2/5-3/7-1/1 | IITA | Tolerant | Susceptible |
| 7 | TZEI 441 | (TZEI 11 x TZEI 8) S6 inb 92-5/5-4/5-1/1 | IITA | Tolerant | Susceptible |
| 8 | TZEI 442 | (TZEI 11 x TZEI 8) S6 inb 92-5/5-5/5-1/1 | IITA | Tolerant | Susceptible |
| 9 | TZEI 443 | (TZEI 11 x TZEI 8) S6 inb 95-2/2-1/5-1/1 | IITA | Tolerant | Tolerant |
| 10 | TZEI 449 | (TZEI 11 x TZEI 8) S6 inb 107-6/6-4/7-1/1 | IITA | Tolerant | Tolerant |
| 11 | TZEI 450 | (TZEI 11 x TZEI 8) S6 inb 112-2/4-4/4-1/1 | IITA | Tolerant | Tolerant |
| 12 | TZEI 455 | (TZEI 11 x TZEI 8) S6 inb 133-3/3-3/3-1/1 | IITA | Tolerant | Susceptible |
| 13 | TZEI 461 | (TZEI 11 x TZEI 8) S6 inb 148-1/3-4/6-1/1 | IITA | Tolerant | Susceptible |
| 14 | TZEI 462 | (TZEI 11 x TZEI 8) S6 inb 148-1/3-6/6-1/1 | IITA | Tolerant | Tolerant |
| 15 | TZEI 464 | (TZEI 11 x TZEI 8) S6 inb 148-3/3-3/5-1/1 | IITA | Tolerant | Susceptible |
| 16 | TZEI 465 | (TZEI 11 x TZEI 8) S6 inb 148-3/3-4/5-1/1 | IITA | Tolerant | Tolerant |
| 17 | TZEI 470 | (TZEI 11 x TZEI 8) S6 inb 154-3/3-1/5-1/1 | IITA | Tolerant | Tolerant |
| 18 | TZEI 472 | (TZEI 11 x TZEI 8) S6 inb 154-3/3-4/5-1/1 | IITA | Tolerant | Tolerant |
| 19 | TZEI 474 | (TZEI 11 x TZEI 8) S6 inb 170-1/3-2/6-1/1 | IITA | Tolerant | Tolerant |
| 20 | TZEI 483 | (TZEI 11 x TZEI 8) S6 inb 184-3/3-4/6-1/1 | IITA | Tolerant | Tolerant |
| 21 | TZEI 484 | (TZEI 11 x TZEI 8) S6 inb 184-3/3-6/6-1/1 | IITA | Tolerant | Tolerant |
| 22 | TZEI 486 | (TZEI 11 x TZEI 8) S6 inb 185-1/2-4/5-1/1 | IITA | Tolerant | Tolerant |
| 23 | TZEI 494 | (TZEI 11 x TZEI 8) S6 inb 201-1/2-3/6-1/1 | IITA | Susceptible | Tolerant |
| 24 | TZEI 495 | (TZEI 11 x TZEI 8) S6 inb 201-1/2-4/6-1/1 | IITA | Tolerant | Tolerant |
| 25 | TZEI 507 | (TZEI 11 x TZEI 8) S6 inb 238-3/4-5/6-1/1 | IITA | Tolerant | Tolerant |
| 26 | TZEI 508 | (TZEI 11 x TZEI 8) S6 inb 248-1/4-1/4-1/1 | IITA | Tolerant | Susceptible |
| 27 | TZEI 515 | (TZEI 11 x TZEI 8) S6 inb 258-1/4-2/6-1/1 | IITA | Tolerant | Susceptible |
| 28 | TZEI 516 | (TZEI 11 x TZEI 8) S6 inb 258-1/4-5/6-1/1 | IITA | Tolerant | Tolerant |
| 29 | TZEI 518 | (TZEI 11 x TZEI 8) S6 inb 258-2/4-5/7-1/1 | IITA | Tolerant | Tolerant |
| 30 | TZEI 520 | (TZEI 11 x TZEI 8) S6 inb 263-1/2-2/6-1/1 | IITA | Tolerant | Tolerant |
| 31 | TZEI 522 | (TZEI 11 x TZEI 8) S6 inb 282-2/3-1/5-1/1 | IITA | Tolerant | Tolerant |
| 32 | TZEI 124 | TZE-Y Pop STR Co S6 Inbred 3-1-3 | IITA | Susceptible | Tolerant |
| 33 | TZEI 16 | TZE Comp5-Y C6 S6 Inbred 31 | IITA | Tolerant | Susceptible |
| 34 | TZEI 24 | TZE-Y Pop STR Co S6 Inbred 142-2-2 | IITA | Tolerant | Resistant |
| 35 | TZEI 160 | TZE-Y Pop STR Co S6 Inbred 102-2-3 | IITA | Tolerant | Tolerant |
| 36 | TZEI 161 | TZE-Y Pop STR Co S6 Inbred 103-2-3 | IITA | Tolerant | Tolerant |
| 37 | TZEI 173 | TZE Comp5-Y C6 S6 Inbred 21A | IITA | Susceptible | Tolerant |
| 38 | TZEI 175 | TZE Comp5-Y C6 S6 Inbred 25B | IITA | Susceptible | Tolerant |
| 39 | TZEI 182 | TZE-Y Pop STR Co S6 Inbred 152-2-2 | IITA | Tolerant | Tolerant |
| 40 | ENT 8 | [M37W/ZM607#bF37sr-2-3sr-6-2-X]-8-2-X-1-BB | CIMMYT | Tolerant | Susceptible |
| 41 | ENT 17 | [(87036/87923)-X-800-3-1-X-1-B-B-1-1-1-B-B-xP | CIMMYT | Tolerant | Tolerant |
| 42 | TZEI 23 (Tester) | TZE-Y Pop STR Co S6 Inbred 62-2-3 | IITA | Tolerant | Resistant |
| 43 | TZEI 129 (Tester) | TZE-Y Pop STR Co S6 Inbred 16-1-3 | IITA | Tolerant | Susceptible |
| 44 | TZEI 10 (Tester) | TZE-Y Pop STR Co S6 Inbred 152 | IITA | Tolerant | Tolerant |
| 45 | TZEI 17 (Tester) | TZE Comp5-Y C6 S6 Inbred 35 | IITA | Tolerant | Tolerant |
| 46 | ENT 13 (Tester) | [M37W/ZM607#bF37sr-2-3sr-6-2-X]-8-2-X-1-BB- | CIMMYT | Tolerant | Susceptible |

Supplementary Table 2. Environments, locations, agro-ecological zone, research conditions, years and mean grain yield of early maturing maize hybrids evaluated in Nigeria.

| Env. | Location | AEZ | Research condition | Year | Grain yield (kg ha-1) |
| --- | --- | --- | --- | --- | --- |
| 1 | Minjibir (12° 00’N, 8°22’E, 445 m asl, and 800 mm annual rainfall) | SS | Induced drought | 2014 | 1782 |
| 2 | Ikenne (6^о^ 53’N, 30^о^ 42’E, 60 m asl, 1200 mm annual rainfall) | FST | Induced drought | 2015 | 1774 |
| 3 | Kadawa (12°11’N, 8°37’E, 580 m asl, 800 mm annual rainfall) | SS | Terminal drought | 2016 | 3360 |
| 4 | Mokwa (9^о^18’N, 5^о^ 4’E, 457 m asl, 1100 mm annual rainfall) | SGS | Low N | 2015 | 3404 |
| 5 | Ile-Ife (7° 28’ N, 4° 33’ E, 244 m asl, 1200 mm annual rainfall) | FST | Low N | 2015 | 3220 |
| 6 | Mokwa (9^о^18’N, 5^о^ 4’E, 457 m asl, 1100 mm annual rainfall) | SGS | Low N | 2016 | 1489 |
| 7 | Mokwa (9^о^18’N, 5^о^ 4’E, 457 m m asl, 1100 mm annual rainfall) | SGS | *Striga* infestation | 2015 | 2602 |
| 8 | Abuja (9^о^ 16’N, 7^о^ 20’E, 300 m asl, 1500 mm annual rainfall) | SGS | *Striga* infestation | 2015 | 2815 |
| 9 | Abuja (9^о^ 16’N, 7^о^ 20’E, 300 m asl, 1500 mm annual rainfall) | SGS | *Striga* infestation | 2016 | 4426 |
| 10 | Mokwa (9^о^18’N, 5^о^ 4’E, 457 m asl, 1100 mm annual rainfall) | SGS | *Striga* infestation | 2016 | 2651 |
| 11 | Mokwa (9^о^18’N, 5^о^ 4’E, 457 m asl, 1100 mm annual rainfall) | SGS | Optimal | 2015 | 6250 |
| 12 | Mokwa (9^о^18’N, 5^о^ 4’E, 457 m asl, 1100 mm annual rainfall) | SGS | Optimal | 2016 | 3707 |
| 13 | Ikenne (6^о^ 53’N, 30^о^ 42’E, 60 m asl, 1200 mm annual rainfall) | FST | Optimal | 2015 | 4670 |
| 14 | Ikenne (6^о^ 53’N, 30^о^ 42’E, 60 m asl, 1200 mm annual rainfall) | FST | Optimal | 2016 | 4048 |
| 15 | Abuja (9^о^ 16’N, 7^о^ 20’E, 300 m asl, 1500 mm annual rainfall) | SGS | Optimal | 2015 | 5208 |
| 16 | Abuja (9^о^ 16’N, 7^о^ 20’E, 300 m asl, 1500 mm annual rainfall) | SGS | Optimal | 2016 | 6001 |
| 17 | Ile-Ife (7° 28’ N, 4° 33’ E, 244 m asl, 1200 mm annual rainfall) | FST | Optimal | 2015 | 4446 |

Env. = Environment; AEZ = Agro-ecological zone; SS = Sudan savanna; SGS = Southern guinea savanna; FST = Forest–savanna transition zone.

Supplementary Table 3. Grain yield and other agronomic traits of early maturing maize hybrids (the best 15 and the worst 10 based on base index) and five checks evaluated under drought (DT) and optimal (OP) environments in 2015 and 2016.

| **Hybrid** | **YIELD** | |  | **POLLEN** | **DYSK** | **ASI** | **PLHT** | **PASP** | **EASP** | **EPP** | **STGR** | **YRD** | **DTBI** |
| --- | --- | --- | --- | --- | --- | --- | --- | --- | --- | --- | --- | --- | --- |
|  | **DT** | OP |  | **DT** | | | | | | | | | |
| TZEI 443 x ENT 13 | 4967 | 7006 |  | 55 | 59 | 3.12 | 166.22 | 4.57 | 3.71 | 0.98 | 3.31 | 29.10 | 13.50 |
| TZEI 515 x ENT 13 | 4091 | 5785 |  | 55 | 57 | 1.32 | 156.74 | 4.92 | 4.27 | 0.94 | 2.90 | 29.28 | 11.69 |
| ENT 8 x TZEI 23 | 4594 | 5470 |  | 52 | 54 | 1.74 | 153.75 | 5.29 | 4.43 | 0.94 | 3.07 | 16.01 | 11.37 |
| TZEI 160 x TZEI 10 | 3913 | 4619 |  | 55 | 56 | 1.67 | 149.39 | 4.57 | 4.33 | 0.92 | 2.88 | 15.30 | 11.15 |
| TZEI 182 x ENT 13 | 3622 | 6263 |  | 55 | 57 | 1.95 | 164.11 | 4.79 | 4.48 | 0.93 | 2.35 | 42.17 | 10.19 |
| TZEI 470 x ENT 13 | 3604 | 5769 |  | 55 | 57 | 2.18 | 168.13 | 4.45 | 4.40 | 1.00 | 3.17 | 37.53 | 9.95 |
| TZEI 515 x TZEI 129 | 4013 | 5415 |  | 55 | 57 | 1.33 | 157.13 | 4.93 | 4.23 | 0.84 | 3.64 | 25.89 | 9.25 |
| TZEI 175 x TZEI 23 | 3802 | 5519 |  | 56 | 57 | 1.83 | 144.80 | 5.08 | 4.30 | 0.88 | 2.97 | 31.11 | 9.11 |
| TZEI 507 x TZEI 129 | 3948 | 5013 |  | 55 | 57 | 2.23 | 158.18 | 5.22 | 5.05 | 1.07 | 3.12 | 21.25 | 8.93 |
| TZEI 518 x TZEI 17 | 3643 | 5552 |  | 57 | 59 | 1.58 | 134.47 | 5.30 | 4.86 | 1.03 | 2.98 | 34.38 | 8.88 |
| TZEI 24 x TZEI 17 | 3548 | 4446 |  | 55 | 57 | 1.11 | 131.33 | 5.26 | 4.25 | 0.85 | 2.91 | 20.20 | 8.74 |
| TZEI 160 x TZEI 17 | 3651 | 4416 |  | 55 | 57 | 1.37 | 134.66 | 5.44 | 4.67 | 1.00 | 3.12 | 17.32 | 8.74 |
| TZEI 516 x ENT 13 | 3700 | 5537 |  | 57 | 60 | 2.39 | 150.23 | 5.03 | 4.67 | 0.93 | 2.61 | 33.18 | 8.59 |
| TZEI 161 x TZEI 17 | 3386 | 5067 |  | 54 | 55 | 0.86 | 144.82 | 5.10 | 4.72 | 0.90 | 2.93 | 33.19 | 8.42 |
| TZEI 474 x TZEI 17 | 3232 | 4604 |  | 55 | 57 | 1.27 | 140.39 | 4.88 | 4.50 | 0.87 | 2.94 | 29.80 | 8.02 |
| ENT 17 x TZEI 129 | 2161 | 4944 |  | 57 | 61 | 4.10 | 149.44 | 6.09 | 5.78 | 0.66 | 4.60 | 56.30 | -8.25 |
| TZEI 175 x TZEI 10 | 1663 | 4797 |  | 57 | 61 | 4.33 | 126.33 | 5.75 | 5.71 | 0.66 | 4.30 | 65.34 | -8.76 |
| TZEI 450 x TZEI 17 | 1385 | 3245 |  | 57 | 59 | 2.09 | 103.05 | 6.75 | 6.03 | 0.79 | 4.67 | 57.33 | -8.79 |
| TZEI 465 x TZEI 23 | 1068 | 4166 |  | 56 | 60 | 3.77 | 149.68 | 4.51 | 6.40 | 0.60 | 4.86 | 74.35 | -10.19 |
| TZEI 449 x TZEI 23 | 1634 | 4156 |  | 57 | 61 | 3.25 | 110.96 | 6.56 | 6.26 | 0.65 | 4.32 | 60.69 | -10.30 |
| TZEI 470 x TZEI 17 | 1085 | 4274 |  | 58 | 61 | 2.84 | 114.42 | 6.81 | 6.48 | 0.80 | 4.22 | 74.61 | -10.72 |
| TZEI 439 x TZEI 23 | 1588 | 2900 |  | 55 | 59 | 3.66 | 128.97 | 6.13 | 6.65 | 0.67 | 4.92 | 45.24 | -11.64 |
| TZEI 441 x TZEI 23 | 990 | 3992 |  | 56 | 58 | 2.07 | 123.19 | 6.87 | 6.28 | 0.71 | 5.18 | 75.20 | -12.31 |
| TZEI 432 x TZEI 10 | 865 | 3521 |  | 64 | 67 | 3.02 | 109.64 | 7.23 | 7.02 | 0.83 | 4.07 | 75.44 | -12.87 |
| TZEI 415 x ENT 13 | 1286 | 5022 |  | 58 | 63 | 5.14 | 150.40 | 5.94 | 6.42 | 0.59 | 4.26 | 74.39 | -13.10 |
| Check 1 | 3500 | 7260 |  | 55 | 58 | 3.01 | 159.66 | 4.52 | 4.68 | 0.78 | 3.14 | 51.79 | 5.91 |
| Check 2 | 2869 | 5419 |  | 57 | 60 | 3.29 | 142.85 | 5.23 | 5.41 | 0.77 | 3.45 | 47.07 | 0.26 |
| Check 3 | 3179 | 5092 |  | 55 | 58 | 3.28 | 132.78 | 5.42 | 5.60 | 0.79 | 4.03 | 37.58 | -0.38 |
| Check 4 | 2944 | 5930 |  | 58 | 60 | 2.07 | 163.47 | 4.95 | 5.10 | 0.83 | 4.01 | 50.35 | 2.69 |
| Check 5 | 2915 | 6487 |  | 56 | 60 | 3.56 | 144.24 | 5.13 | 5.30 | 0.79 | 3.83 | 55.07 | 0.01 |
| **Mean** | **2639** | **4904** |  | **56** | **59** | **2.46** | **139.15** | **5.70** | **5.33** | **0.84** | **3.73** | **45.33** |  |
| **SED** | **382** | **251** |  | **1** | **1** | **0.70** | **7.13** | **0.38** | **0.37** | **0.08** | **0.49** |  |  |

YIELD = Grain yield (kg ha^-1^); Pollen= days to 50% anthesis ; DYSK = days to 50% silking; ASI = anthesis-silking interval; PLHT = plant height (cm); PASP = plant aspect (1-9); EASP = ear aspect (1-9); EPP =ears per plant; STGR= stay-green characteristic (1 - 9); YRD = yield reduction (%); DT BI= drought base index; Check 1 = TZEI 124 x TZEI 25; Check 2 = TZEI 11 x TZEI 24; Check 3 = (TZEI 135 x TZEI 157) x TZEI 17; Check 4 = TZE-Y Pop DT STR x TZEI 13; Check 5 = TZE-Y Pop DT STR x TZEI 17

Supplementary Table 4. Grain yield and other agronomic traits of early maturing maize hybrids (the best 15 and the worst 10 based on base index) and five checks evaluated under low N (LN) and optimal (OP) environments in 2015 and 2016.

| **Hybrid** | **YIELD** | |  | **POLLEN** | **DYSK** | **ASI** | **PLHT** | **PASP** | **EASP** | **EPP** | **STGR** | **YRD** | **LNBI** |
| --- | --- | --- | --- | --- | --- | --- | --- | --- | --- | --- | --- | --- | --- |
|  | **LN** | OP |  | **LN** | | | | | | | | | |
| TZEI 443 x ENT 13 | 4096 | 7006 |  | 53 | 54 | 0.69 | 153.05 | 3.89 | 4.38 | 0.99 | 2.81 | 41.54 | 10.18 |
| TZEI 462 x TZEI 17 | 3829 | 5996 |  | 54 | 54 | 0.14 | 136.74 | 5.46 | 4.35 | 1.01 | 1.96 | 36.14 | 10.08 |
| ENT 8 x TZEI 10 | 3980 | 6217 |  | 52 | 53 | 0.33 | 143.98 | 4.59 | 4.30 | 1.01 | 3.03 | 35.99 | 9.43 |
| ENT 8 x TZEI 17 | 3923 | 6615 |  | 53 | 53 | 0.00 | 134.16 | 4.92 | 4.86 | 0.93 | 3.07 | 40.71 | 7.71 |
| TZEI 455 x ENT 13 | 3916 | 5446 |  | 51 | 52 | 0.56 | 155.47 | 4.07 | 4.75 | 0.97 | 3.43 | 28.11 | 7.60 |
| TZEI 461 x TZEI 10 | 3630 | 5272 |  | 53 | 53 | 0.04 | 142.23 | 4.88 | 4.77 | 1.00 | 3.08 | 31.15 | 7.46 |
| TZEI 465 x ENT 13 | 3765 | 6601 |  | 52 | 52 | 0.46 | 146.96 | 4.66 | 4.71 | 0.92 | 3.13 | 42.97 | 6.57 |
| TZEI 443 x TZEI 17 | 3299 | 5707 |  | 54 | 54 | 0.37 | 130.89 | 4.79 | 4.78 | 0.98 | 2.75 | 42.19 | 6.33 |
| TZEI 16 x TZEI 23 | 3601 | 5626 |  | 51 | 52 | 1.28 | 134.46 | 4.57 | 4.28 | 0.99 | 2.91 | 36.00 | 6.27 |
| TZEI 462 x TZEI 10 | 3594 | 6538 |  | 53 | 54 | 0.97 | 139.11 | 5.24 | 4.78 | 1.01 | 2.48 | 45.02 | 6.12 |
| TZEI 516 x ENT 13 | 3598 | 5537 |  | 52 | 52 | 0.51 | 135.52 | 4.59 | 4.92 | 0.91 | 3.19 | 35.02 | 5.48 |
| ENT 8 x TZEI 23 | 3515 | 5470 |  | 50 | 51 | 0.29 | 133.67 | 4.98 | 5.42 | 1.04 | 3.29 | 35.73 | 5.41 |
| TZEI 474 x TZEI 17 | 3176 | 4604 |  | 54 | 54 | 0.41 | 130.43 | 5.08 | 5.22 | 1.02 | 2.60 | 31.01 | 5.35 |
| TZEI 515 x TZEI 129 | 3241 | 5415 |  | 51 | 52 | 0.20 | 150.42 | 4.60 | 4.82 | 0.89 | 3.14 | 40.14 | 5.08 |
| ENT 17 x TZEI 10 | 3500 | 5253 |  | 53 | 54 | 1.14 | 131.11 | 4.50 | 5.11 | 0.97 | 2.83 | 33.37 | 5.01 |
| TZEI 518 x TZEI 23 | 1780 | 3107 |  | 51 | 52 | 1.06 | 119.84 | 6.13 | 6.43 | 0.99 | 3.60 | 42.71 | -6.39 |
| TZEI 428 x TZEI 129 | 1885 | 4457 |  | 52 | 54 | 1.52 | 147.84 | 5.25 | 5.70 | 0.88 | 3.97 | 57.71 | -6.43 |
| TZEI 432 x TZEI 10 | 1561 | 3521 |  | 55 | 56 | 0.68 | 129.63 | 5.66 | 6.08 | 0.75 | 3.19 | 55.66 | -6.58 |
| TZEI 516 x TZEI 23 | 1508 | 2900 |  | 52 | 53 | 1.58 | 133.64 | 6.35 | 6.53 | 1.02 | 2.93 | 47.99 | -7.26 |
| TZEI 16 x TZEI 17 | 2051 | 4447 |  | 55 | 57 | 1.56 | 119.28 | 6.03 | 6.02 | 0.79 | 3.40 | 53.87 | -7.34 |
| TZEI 433 x TZEI 23 | 1566 | 3317 |  | 52 | 53 | 0.93 | 106.51 | 6.46 | 6.43 | 0.95 | 3.64 | 52.80 | -7.77 |
| TZEI 432 x TZEI 17 | 2214 | 3535 |  | 55 | 57 | 2.49 | 130.50 | 5.61 | 5.72 | 0.70 | 3.43 | 37.37 | -8.57 |
| TZEI 182 x TZEI 10 | 1469 | 2705 |  | 53 | 54 | 1.08 | 122.40 | 6.11 | 6.49 | 0.86 | 3.50 | 45.67 | -8.59 |
| TZEI 439 x TZEI 23 | 1589 | 2900 |  | 53 | 54 | 1.09 | 117.47 | 6.33 | 6.07 | 0.84 | 3.84 | 45.20 | -8.79 |
| TZEI 522 x TZEI 23 | 1812 | 3689 |  | 51 | 53 | 1.75 | 126.19 | 6.14 | 6.45 | 0.90 | 3.68 | 50.88 | -8.88 |
| Check 1 | 2834 | 7260 |  | 53 | 55 | 1.17 | 153.42 | 4.60 | 4.87 | 0.87 | 2.74 | 60.97 | 2.17 |
| Check 2 | 3038 | 5419 |  | 52 | 53 | 1.08 | 131.99 | 4.98 | 5.13 | 0.92 | 3.19 | 43.94 | 1.62 |
| Check 3 | 2882 | 5092 |  | 53 | 53 | 0.57 | 132.30 | 4.92 | 5.83 | 0.83 | 3.33 | 43.40 | 0.06 |
| Check 4 | 2881 | 5930 |  | 54 | 55 | 0.95 | 143.15 | 4.88 | 5.06 | 0.78 | 3.93 | 51.42 | -1.23 |
| Check 5 | 3411 | 6487 |  | 52 | 53 | 0.60 | 140.48 | 4.91 | 4.93 | 0.84 | 3.36 | 47.42 | 3.22 |
| **Mean** | **2704** | **4904** |  | **53** | **53** | **0.79** | **135.09** | **5.24** | **5.42** | **0.92** | **3.36** | **44.49** |  |
| **SED** | **236** | **251** |  | **1** | **1** | **0.31** | **6.60** | **0.28** | **0.27** | **0.05** | **0.29** |  |  |

YIELD = Grain yield (kg ha^-1^); Pollen= days to 50% anthesis ; DYSK = days to 50% silking; ASI = anthesis-silking interval; PLHT = plant height (cm); PASP = plant aspect (1-9); EASP = ear aspect (1-9); EPP =ears per plant; STGR= stay-green characteristic (1 - 9); YRD = yield reduction (%); LNBI= low N base index; Check 1 = TZEI 124 x TZEI 25; Check 2 = TZEI 11 x TZEI 24; Check 3 = (TZEI 135 x TZEI 157) x TZEI 17; Check 4 = TZE-Y Pop DT STR x TZEI 13; Check 5 = TZE-Y Pop DT STR x TZEI 17

Supplementary Table 5. Grain yield and other agronomic traits of early maturing maize hybrids (the best 15 and the worst 10 based on base index) and five checks evaluated under *Striga-*infested (ST) and optimal (OP) environments in 2015 and 2016.

| **Hybrids** | **YIELD** | |  | **POLLEN** | **DYSK** | **ASI** | **PLHT** | **EASP** | **EPP** | **SDR8** | **SDR10** | **ESP8** | **ESP10** | **YRD** | **STBI** |
| --- | --- | --- | --- | --- | --- | --- | --- | --- | --- | --- | --- | --- | --- | --- | --- |
|  | **ST** | OP |  | ST | | | | | | | | | | | |
| TZEI 472 x ENT 13 | 4479 | 6012 |  | 53 | 55 | 1.95 | 145.39 | 4.59 | 0.92 | 3.39 | 4.25 | 10.98 | 14.97 | 25.50 | 10.06 |
| TZEI 470 x ENT 13 | 3948 | 5769 |  | 56 | 57 | 0.89 | 140.15 | 5.00 | 0.92 | 3.31 | 4.48 | 4.14 | 12.23 | 31.57 | 9.01 |
| TZEI 160 x TZEI 10 | 3988 | 4619 |  | 53 | 54 | 1.16 | 139.75 | 5.05 | 0.99 | 3.66 | 4.31 | 10.75 | 11.77 | 13.66 | 8.85 |
| TZEI 24 x TZEI 129 | 4078 | 5325 |  | 54 | 55 | 0.69 | 148.54 | 4.64 | 0.89 | 3.70 | 4.27 | 5.33 | 8.56 | 23.42 | 8.83 |
| TZEI 462 x TZEI 17 | 4065 | 5996 |  | 55 | 57 | 1.09 | 154.79 | 4.65 | 1.07 | 4.20 | 4.82 | 8.91 | 10.01 | 32.21 | 7.79 |
| TZEI 175 x TZEI 23 | 4043 | 5519 |  | 54 | 56 | 2.22 | 137.94 | 4.73 | 0.99 | 4.36 | 4.61 | 6.74 | 8.54 | 26.74 | 7.21 |
| TZEI 24 x TZEI 10 | 3768 | 4205 |  | 54 | 55 | 1.48 | 136.14 | 5.15 | 0.92 | 4.11 | 4.32 | 5.12 | 6.95 | 10.39 | 7.19 |
| TZEI 415 x TZEI 23 | 3786 | 3869 |  | 52 | 53 | 1.13 | 140.71 | 4.91 | 1.00 | 3.78 | 4.49 | 11.88 | 15.25 | 2.14 | 7.06 |
| TZEI 161 x TZEI 10 | 3937 | 5331 |  | 52 | 53 | 0.62 | 130.01 | 4.98 | 0.95 | 3.91 | 4.72 | 8.56 | 9.38 | 26.15 | 7.06 |
| TZEI 455 x TZEI 10 | 3584 | 4676 |  | 53 | 55 | 1.54 | 145.83 | 4.73 | 1.00 | 3.96 | 4.76 | 4.80 | 5.82 | 23.36 | 7.05 |
| TZEI 455 x TZEI 17 | 3948 | 4232 |  | 54 | 55 | 1.20 | 151.65 | 4.91 | 1.01 | 4.41 | 4.62 | 4.16 | 11.03 | 6.71 | 7.01 |
| TZEI 462 x ENT 13 | 4614 | 6829 |  | 55 | 59 | 3.13 | 143.11 | 5.04 | 0.93 | 4.02 | 4.90 | 13.54 | 18.25 | 32.44 | 6.81 |
| TZEI 507 x TZEI 10 | 3670 | 4030 |  | 54 | 55 | 1.56 | 130.26 | 4.96 | 1.01 | 4.18 | 4.58 | 5.53 | 9.32 | 8.93 | 6.80 |
| TZEI 483 x TZEI 10 | 3725 | 4852 |  | 52 | 53 | 1.16 | 136.49 | 5.47 | 1.00 | 4.08 | 4.73 | 7.46 | 8.92 | 23.23 | 6.62 |
| TZEI 443 x ENT 13 | 4466 | 7006 |  | 54 | 56 | 1.45 | 137.32 | 4.78 | 0.87 | 4.06 | 4.49 | 13.95 | 16.53 | 36.26 | 6.58 |
| ENT 17 x TZEI 10 | 2252 | 5253 |  | 55 | 57 | 2.68 | 128.84 | 5.61 | 0.77 | 4.95 | 5.87 | 16.29 | 18.07 | 57.12 | -7.96 |
| TZEI 124 x TZEI 17 | 2457 | 5159 |  | 56 | 58 | 2.18 | 144.09 | 5.78 | 0.64 | 5.17 | 5.89 | 9.43 | 10.96 | 52.37 | -8.13 |
| TZEI 449 x TZEI 23 | 2126 | 4156 |  | 55 | 58 | 2.03 | 129.27 | 6.12 | 0.76 | 5.40 | 6.10 | 10.15 | 14.90 | 48.86 | -9.25 |
| TZEI 455 x TZEI 23 | 1333 | 2428 |  | 54 | 55 | 1.45 | 129.39 | 6.35 | 0.89 | 5.46 | 6.11 | 7.09 | 6.16 | 45.12 | -9.29 |
| TZEI 520 x TZEI 17 | 2144 | 4574 |  | 57 | 59 | 2.69 | 128.41 | 5.79 | 0.79 | 5.35 | 5.87 | 20.35 | 20.55 | 53.13 | -9.88 |
| TZEI 449 x TZEI 17 | 2538 | 4769 |  | 56 | 59 | 3.15 | 128.73 | 5.88 | 0.73 | 5.30 | 6.02 | 18.80 | 26.29 | 46.79 | -9.94 |
| ENT 17 x TZEI 17 | 2012 | 4526 |  | 57 | 59 | 2.64 | 127.96 | 6.34 | 0.70 | 5.19 | 6.05 | 14.50 | 16.87 | 55.55 | -10.43 |
| TZEI 450 x TZEI 17 | 2140 | 3245 |  | 56 | 57 | 3.22 | 138.33 | 5.89 | 0.76 | 5.50 | 6.40 | 14.99 | 18.30 | 34.07 | -11.16 |
| TZEI 16 x TZEI 17 | 1995 | 4447 |  | 59 | 62 | 2.68 | 114.02 | 6.09 | 0.67 | 5.33 | 6.22 | 12.54 | 14.65 | 55.13 | -11.23 |
| TZEI 449 x TZEI 129 | 2268 | 5134 |  | 57 | 59 | 1.40 | 142.00 | 6.11 | 0.65 | 5.62 | 6.14 | 13.87 | 18.28 | 55.82 | -11.57 |
| Check 1 | 4157 | 7260 |  | 54 | 55 | 1.13 | 148.15 | 5.09 | 0.81 | 4.15 | 5.14 | 8.45 | 11.07 | 42.74 | 4.24 |
| Check 2 | 4799 | 5419 |  | 55 | 58 | 2.97 | 138.22 | 4.77 | 0.91 | 3.93 | 4.32 | 6.49 | 10.83 | 11.46 | 10.33 |
| Check 3 | 2962 | 5092 |  | 54 | 55 | 1.98 | 150.15 | 5.27 | 0.86 | 4.81 | 5.35 | 14.61 | 14.33 | 41.84 | -2.37 |
| Check 4 | 2665 | 5930 |  | 58 | 60 | 2.66 | 151.48 | 5.76 | 0.76 | 4.84 | 5.66 | 14.45 | 24.93 | 55.07 | -6.51 |
| Check 5 | 3120 | 6487 |  | 55 | 57 | 1.58 | 130.69 | 5.38 | 0.73 | 4.78 | 5.67 | 16.87 | 22.59 | 51.90 | -5.19 |
| Mean | 3124 | 4904 |  | 55 | 56 | 1.81 | 138.66 | 5.37 | 0.88 | 4.55 | 5.25 | 9.51 | 12.95 | 35.35 |  |
| **SED** | 370 | 251 |  | 1 | 1 | 0.52 | 6.62 | 0.26 | 0.05 | 0.26 | 0.27 | 3.13 | 3.25 |  |  |

YIELD = Grain yield (kg ha^-1^); Pollen= days to 50% anthesis ; DYSK = days to 50% silking; ASI = anthesis-silking interval; PLHT = plant height (cm); EASP = ear aspect (1-9); EPP =ears per plant; SDR8 and SDR10 = *Striga* damage at 8 and 10 WAP; ESP8 and ESP10 = number of emerged *Striga* plants at 8 and 10 WAP; YRD = yield reduction (%); STBI= *Striga* base index; Check 1 = TZEI 124 x TZEI 25; Check 2 = TZEI 11 x TZEI 24; Check 3 = (TZEI 135 x TZEI 157) x TZEI 17; Check 4 = TZE-Y Pop DT STR x TZEI 13; Check 5 = TZE-Y Pop DT STR x TZEI 17

Supplementary Table 6. Grain yield and other agronomic traits of early maturing maize hybrids (the best 15 and the

worst 10 multiple-stress based on base index) and five checks evaluated under optimal environments in 2015 and 2016.

| **Hybrid** | **YIELD** | **POLLEN** | **DYSK** | **ASI** | **PLHT** | **PASP** | **EASP** | **EPP** | **MBI** |
| --- | --- | --- | --- | --- | --- | --- | --- | --- | --- |
| TZEI 443 x ENT 13 | 7006 | 52.71 | 53.15 | 0.44 | 164.21 | 3.67 | 3.72 | 1.02 | 17.46 |
| TZEI 470 x ENT 13 | 5769 | 51.35 | 52.61 | 1.37 | 163.33 | 4.52 | 4.69 | 0.98 | 13.63 |
| TZEI 160 x TZEI 10 | 4619 | 50.97 | 51.32 | 0.33 | 149.69 | 5.07 | 5.10 | 0.95 | 13.32 |
| TZEI 175 x TZEI 23 | 5519 | 51.39 | 51.71 | 0.66 | 149.01 | 5.14 | 5.21 | 1.05 | 11.77 |
| TZEI 161 x TZEI 10 | 5331 | 50.75 | 50.88 | 0.14 | 150.07 | 5.02 | 4.64 | 0.95 | 11.74 |
| TZEI 462 x TZEI 17 | 5996 | 53.07 | 53.14 | 0.48 | 154.81 | 5.22 | 4.39 | 0.96 | 10.02 |
| TZEI 24 x TZEI 129 | 5325 | 51.87 | 51.97 | 0.35 | 162.37 | 4.60 | 4.56 | 0.89 | 9.60 |
| TZEI 507 x TZEI 129 | 5013 | 51.46 | 52.08 | 0.58 | 159.21 | 4.73 | 4.77 | 0.95 | 9.42 |
| TZEI 516 x ENT 13 | 5537 | 53.02 | 53.68 | 0.66 | 153.27 | 5.04 | 4.92 | 0.96 | 9.30 |
| TZEI 472 x ENT 13 | 6012 | 51.66 | 51.94 | 0.32 | 163.57 | 4.15 | 4.38 | 0.96 | 9.04 |
| TZEI 462 x TZEI 10 | 6538 | 52.78 | 52.97 | 0.57 | 168.93 | 4.80 | 4.34 | 1.00 | 8.82 |
| TZEI 518 x TZEI 17 | 5552 | 53.08 | 53.17 | 0.88 | 147.05 | 5.24 | 4.95 | 0.91 | 8.45 |
| TZEI 415 x TZEI 23 | 3869 | 51.07 | 51.55 | 0.49 | 135.70 | 5.64 | 5.33 | 0.95 | 8.43 |
| TZEI 455 x TZEI 17 | 4232 | 54.71 | 54.75 | 0.12 | 138.41 | 5.91 | 5.30 | 1.03 | 8.19 |
| TZEI 455 x ENT 13 | 5446 | 52.06 | 52.35 | 0.23 | 160.85 | 4.74 | 4.88 | 0.95 | 7.67 |
| TZEI 520 x TZEI 17 | 4574 | 53.32 | 53.45 | 0.39 | 138.08 | 5.36 | 5.21 | 0.91 | -10.50 |
| ENT 8 x ENT 13 | 4841 | 52.45 | 52.79 | 0.49 | 163.35 | 5.22 | 5.43 | 0.97 | -10.89 |
| TZEI 449 x TZEI 17 | 4769 | 53.97 | 54.60 | 0.59 | 142.38 | 5.67 | 4.66 | 0.94 | -11.00 |
| TZEI 470 x TZEI 17 | 4274 | 52.83 | 53.05 | 0.42 | 143.95 | 5.15 | 5.36 | 0.92 | -11.33 |
| TZEI 455 x TZEI 23 | 2428 | 51.95 | 51.96 | 0.19 | 134.34 | 6.30 | 6.27 | 0.96 | -11.54 |
| TZEI 432 x TZEI 10 | 3521 | 61.16 | 63.70 | 2.53 | 122.08 | 5.94 | 5.54 | 0.85 | -11.74 |
| ENT 17 x TZEI 17 | 4526 | 53.31 | 53.71 | 0.40 | 141.13 | 5.64 | 5.44 | 0.89 | -13.09 |
| TZEI 449 x TZEI 23 | 4156 | 52.25 | 52.71 | 0.40 | 143.23 | 5.64 | 5.59 | 0.92 | -13.30 |
| TZEI 16 x TZEI 17 | 4447 | 54.85 | 55.76 | 0.95 | 135.84 | 5.60 | 5.41 | 1.01 | -16.39 |
| TZEI 450 x TZEI 17 | 3245 | 53.27 | 53.61 | 0.50 | 136.74 | 5.89 | 5.85 | 0.83 | -17.99 |
| Check 1 | 7260 | 51.83 | 52.42 | 0.53 | 171.49 | 3.81 | 3.95 | 1.00 | 8.60 |
| Check 2 | 5419 | 51.58 | 52.19 | 0.66 | 150.41 | 4.98 | 5.06 | 0.96 | 8.43 |
| Check 3 | 5092 | 51.82 | 51.76 | 0.12 | 148.35 | 4.94 | 5.14 | 0.84 | -2.59 |
| Check 4 | 5930 | 53.09 | 53.81 | 0.93 | 161.62 | 4.44 | 4.90 | 0.97 | -5.68 |
| Check 5 | 6487 | 51.16 | 51.18 | 0.20 | 158.68 | 4.26 | 4.31 | 1.00 | -2.97 |
| **Mean** | **4904** | **52.27** | **52.78** | **0.63** | **149.09** | **5.21** | **5.08** | **0.94** |  |
| **SED** | **251** | **0.33** | **0.37** | **0.20** | **3.49** | **0.17** | **0.18** | **0.04** |  |

YIELD = Grain yield (kg ha^-1^); Pollen= days to 50% anthesis ; DYSK = days to 50% silking; ASI = anthesis-silking interval;

PLHT = plant height (cm); PASP = plant aspect (1-9); EASP = ear aspect (1-9); EPP =ears per plant.; MBI= multiple base index;

Check 1 = TZEI 124 x TZEI 25; Check 2 = TZEI 11 x TZEI 24; Check 3 = (TZEI 135 x TZEI 157) x TZEI 17;

Check 4 = TZE-Y Pop DT STR x TZEI 13; Check 5 = TZE-Y Pop DT STR x TZEI 17.

Supplementary Table 7. Grain yield and other agronomic traits of early maturing maize hybrids (the best 15 and the

worst 10 based on base index) and checks evaluated across drought, low N, *Striga*-infested, optimal environments

in 2015 and 2016.

| **Hybrid** | **YIELD** | **POLLEN** | **DYSK** | **ASI** | **PLHT** | **EASP** | **EPP** | **MBI** |
| --- | --- | --- | --- | --- | --- | --- | --- | --- |
| TZEI 443 x ENT 13 | 5535 | 53.63 | 54.82 | 1.19 | 156.27 | 4.08 | 0.97 | 17.46 |
| TZEI 470 x ENT 13 | 4416 | 53.20 | 54.46 | 1.36 | 157.55 | 4.86 | 0.95 | 13.63 |
| TZEI 160 x TZEI 10 | 4037 | 52.06 | 52.94 | 0.82 | 145.66 | 5.05 | 0.94 | 13.32 |
| TZEI 175 x TZEI 23 | 4491 | 53.02 | 53.95 | 1.29 | 143.62 | 4.95 | 1.00 | 11.77 |
| TZEI 161 x TZEI 10 | 4382 | 51.99 | 52.57 | 0.59 | 138.86 | 4.81 | 0.94 | 11.74 |
| TZEI 462 x TZEI 17 | 4509 | 54.67 | 55.47 | 0.91 | 148.64 | 4.66 | 0.95 | 10.02 |
| TZEI 24 x TZEI 129 | 4117 | 53.25 | 54.08 | 0.93 | 156.23 | 4.72 | 0.88 | 9.60 |
| TZEI 507 x TZEI 129 | 4055 | 52.60 | 53.65 | 1.12 | 153.85 | 5.01 | 0.96 | 9.42 |
| TZEI 516 x ENT 13 | 4319 | 54.24 | 55.68 | 1.42 | 149.14 | 4.97 | 0.93 | 9.30 |
| TZEI 472 x ENT 13 | 4414 | 52.84 | 54.08 | 1.27 | 156.89 | 4.60 | 0.92 | 9.04 |
| TZEI 462 x TZEI 10 | 4766 | 53.78 | 54.61 | 0.98 | 159.96 | 4.81 | 0.98 | 8.82 |
| TZEI 518 x TZEI 17 | 4311 | 54.27 | 54.94 | 0.98 | 141.16 | 5.09 | 0.92 | 8.45 |
| TZEI 415 x TZEI 23 | 3409 | 51.93 | 52.70 | 0.79 | 134.21 | 5.20 | 0.93 | 8.43 |
| TZEI 455 x TZEI 17 | 3514 | 54.40 | 55.38 | 0.94 | 136.44 | 5.25 | 0.97 | 8.19 |
| TZEI 455 x ENT 13 | 4295 | 53.06 | 54.13 | 1.02 | 160.03 | 4.96 | 0.97 | 7.67 |
| TZEI 520 x TZEI 17 | 3240 | 54.82 | 55.91 | 1.20 | 132.83 | 5.35 | 0.83 | -10.50 |
| ENT 8 x ENT 13 | 3348 | 54.22 | 55.16 | 1.03 | 149.51 | 5.54 | 0.86 | -10.89 |
| TZEI 449 x TZEI 17 | 3352 | 55.39 | 56.69 | 1.23 | 136.12 | 5.23 | 0.86 | -11.00 |
| TZEI 470 x TZEI 17 | 2885 | 54.69 | 55.87 | 1.27 | 131.76 | 5.73 | 0.88 | -11.33 |
| TZEI 455 x TZEI 23 | 1897 | 52.99 | 53.71 | 0.87 | 125.90 | 6.22 | 0.95 | -11.54 |
| TZEI 432 x TZEI 10 | 2502 | 59.15 | 61.28 | 2.16 | 125.82 | 5.95 | 0.83 | -11.74 |
| ENT 17 x TZEI 17 | 3200 | 55.08 | 56.34 | 1.27 | 132.13 | 5.74 | 0.82 | -13.09 |
| TZEI 449 x TZEI 23 | 2983 | 54.00 | 55.43 | 1.37 | 131.87 | 5.86 | 0.84 | -13.30 |
| TZEI 16 x TZEI 17 | 2900 | 56.60 | 58.38 | 1.70 | 125.00 | 5.81 | 0.86 | -16.39 |
| TZEI 450 x TZEI 17 | 2450 | 54.71 | 55.64 | 1.50 | 127.82 | 5.90 | 0.80 | -17.99 |
| Check 1 | 5085 | 53.22 | 54.43 | 1.22 | 160.72 | 4.51 | 0.89 | 8.60 |
| Check 2 | 4403 | 53.31 | 55.05 | 1.74 | 142.96 | 5.06 | 0.91 | 8.43 |
| Check 3 | 3863 | 52.94 | 53.93 | 1.19 | 143.19 | 5.38 | 0.84 | -2.59 |
| Check 4 | 4097 | 55.18 | 56.63 | 1.54 | 156.30 | 5.16 | 0.86 | -5.68 |
| Check 5 | 4521 | 53.13 | 54.24 | 1.19 | 146.33 | 4.85 | 0.87 | -2.97 |
| **Mean** | 3697 | **53.52** | **54.70** | **1.26** | **142.41** | **5.26** | **0.91** |  |
| **SED** | **157** | **0.26** | **0.33** | **0.20** | **2.73** | **0.12** | **0.03** |  |

YIELD = grain yield (kg ha^-1^); Pollen = days to 50% anthesis ; DYSK = days to 50% silking; ASI = anthesis-silking interval;

PLHT = plant height (cm); PASP = plant aspect (1-9); EASP = ear aspect (1-9); EPP =ears per plant; MBI= multiple base index;

Check 1 = TZEI 124 x TZEI 25; Check 2 = TZEI 11 x TZEI 24; Check 3 = (TZEI 135 x TZEI 157) x TZEI 17;

Check 4 = TZE-Y Pop DT STR x TZEI 13; Check 5 = TZE-Y Pop DT STR x TZEI 17.
